# Workplace health promotion to facilitate physical activity among office workers in Sweden

**DOI:** 10.3389/fpubh.2023.1175977

**Published:** 2023-04-13

**Authors:** Oskar Halling Ullberg, Susanna Toivanen, Annika Tillander, Katarina Bälter

**Affiliations:** ^1^Division of Public Health Sciences, School of Health, Care and Social Welfare, Mälardalen University, Västerås, Sweden; ^2^Department of Public Health Sciences, Stockholm University, Stockholm, Sweden; ^3^Department of Statistics and Machine Learning, Linköping University, Linköping, Sweden; ^4^Department of Medical Epidemiology and Biostatistics, Karolinska Institute, Stockholm, Sweden

**Keywords:** monetary allowance, work-life balance, wellness, workplace health promotion, managers

## Abstract

Office workers spend most of their working time being sedentary, contributing to a sedentary lifestyle that increases the risk of developing disease and disability. A gradual decline in cardiorespiratory fitness among adults, along with increased rate of non-communicable diseases across developed countries, makes the workplace an important opportunity for promoting healthy behaviors. This study aimed to investigate: how office companies in Sweden organize and provide workplace health promotion services related to physical activity; the companies' vision for providing workplace health promotion; and potential facilitators and barriers. Nine informants from eight companies participated in the study, and both qualitative and quantitative data were collected by semi-structured interviews. Informants were selected through purposive sampling in collaboration with eight companies in the office market, including companies that own and develop office buildings, shared workspaces, interior design, sustainable solutions, or consult on issues related to the office sector. The framework method was used to analyze the data in a flexible and systematic way. The results showed that workplace health promotion is implemented to maintain employee health, productivity, and employee branding. Also, a significant number of financial resources, organizational support and office space are devoted to workplace health promotion. Convenience and easy access to storage and fitness facilities are key facilitators. In conclusion, this study highlights the importance of employees' engagement in developing and improving workplace health promotion and addressing work-life balance constraints that hinder a healthy lifestyle. Removing barriers on an organizational level may improve the usage of workplace health promotion related to physical activity among office employees.

## 1. Introduction

Office workers spend 80% of their working time being sedentary (1), contributing to a sedentary lifestyle that increases the risk of developing disease and disability (2). A gradual decline in cardiorespiratory fitness among adults, along with increased rate of non-communicable diseases across developed countries (3–5), makes the workplace an important opportunity for promoting healthy behaviors (6, 7). Examples of efforts are the Workplace Health Model by the Center for Disease Control in the US that highlight the importance of healthy behaviors and wellness of employees, including healthy eating, physical activity, smoking cessation, stress management, and disease screening (8). Moreover, reviews of workplace health promotion confirmed that the implementation of best practice programs decreased absenteeism and employer cost and increased employee productivity and health (7, 9). The European Network for Workplace Health emphasizes the same aspects as the CDC, but adds psychosocial factors, work organization, and the work environment into workplace health promotion programs. It defines workplace health promotion as the combined efforts of society, employers, and employees to improve the health and wellbeing of employees at work (6). These efforts include practices, programs, and polices to make the healthy choices easy (6).

Every day, 5.1 million employees go to work in Sweden (10), and about half of these are office workers doing physically inactive work (11). Individual and group-based wellness programs to promote healthy lifestyles at workplaces in Sweden have been offered since the 1970's (12). Furthermore, in 1988 the Swedish government introduced a monetary allowance, also known as a wellness allowance, to promote health and physical activity in the working population. Although this workplace benefit has been available to companies and their employees for more than 30 years, only sparse data are available about its effect on employees. Previous surveys indicate that about 50% of the employees are offered wellness allowances, and of these, only 60–70% are taking advantage of this benefit (13, 14). This suggests that the benefit is underused and does not reach all employees. Wellness allowance is a tax-free company expense that an employer offers an employee, free of charge for the employee by traditional receipt accounting. The maximum non-taxable amount allowed by the government is currently 450 Euro per employee and year (15), but lower amounts are common and vary from company to company. How the employee can use the wellness allowance is also regulated by the government. Examples of health-promoting activities are gym memberships, ski passes, massages, dietary advice, or smoking cessation. However, the allowance cannot be used for purchase of exercise equipment, education, beauty treatments, or medical expenses. Moreover, the employer may have local regulations regarding how the wellness allowance should be used (15), and it may be provided *via* benefit and rewards platforms. Other health-promoting strategies, in addition to wellness allowances, include in-house fitness facilities, exercise sessions by partner companies, and health education. Many companies also offer a “wellness hour,” i.e., employees may spend 1 h per week during regular working hours on health-promoting activities (15, 16).

### 1.1. Influence of work-related factors on employee physical activity

A physically active lifestyle during and after working hours is associated with improved health behaviors, productivity, work ethic, reduced sickness absence among employees, and positive financial return for employers (7, 17–20). However, workplace policies and norms, full-time workloads, performance concerns, and managers' attitudes may be barriers for the employees to engage in healthy behaviors during working hours (21–23). Other possible barriers are environmental limitations, such as lack of public space for outdoor exercise and indoor facilities for physical activity near the office (21, 24, 25). Workplace health promotion may also be perceived as ethically or morally troublesome by some employees who perceive it as an invasion of privacy (26), and therefore, workplace health promotion should be encouraged respectfully with emphasis on voluntary participation. Previous research from various countries has used interventions to improve physical activity in office workers in the form of fitness programs (27), exercise during paid working hours (28), physically active workstations (29, 30), public policies programs (31), social support (32), sociocultural, and environmental change (33, 34), digital tools (35), motivational materials (36), as well as expert consultations and education (37). All have shown improved physical activity and decreased sedentary time.

Studies of workplace health promotion and wellness allowance usage in Sweden emphasize that organizations need to be able to disseminate information from well-developed policies to improve participation and usage (38, 39). Therefor is it essential to provide managers with adequate time, communication training, and education to be able to promote workplace health (38, 39). To address issues related to work-life balance, such as time constraints experienced by employees with diverse life situations a “one-size-fits-all” kind of approach should be avoided (39). Still, little is known about how private companies in Sweden with mainly office workers implement workplace health promotion to facilitate physical activity in Sweden. The study's purpose was to investigate how office companies organize and provide access to workplace health promotion services related to physical activity and wellness allowance. Its purpose was also to study companies' vision and intention for providing workplace health promotion and to explore barriers encountered.

## 2. Materials and methods

### 2.1. Setting and design

The present study is part of a research project entitled *Concepts for the Sustainable Office of the Future* (SOFCO), exploring how a sustainable lifestyle and health may be supported among office workers in Sweden. The project is done in collaboration with eight companies in the office market, including companies that own and develop office buildings, shared workspaces, interior design, sustainable solutions, or consult on issues related to the office sector, both on a national and international level. The present study employs a qualitative approach to explore how companies describe and finance workplace health promotion for employees, with focus on facilitating, supporting, and encouraging physical activity, both at work and outside of work. The study was approved by the Ethical Review Authority, Sweden (Dnr: 2021-02309).

### 2.2. Participants

The informants compromised of nine persons responsible for workplace health promotion at companies in the office sector. Although the informants' work-places were located in the two biggest cities in Sweden, the information they provided were representative for all employees working at each company including local work-places around the country. Initially 11 personnel were contacted as potential informants, of which nine were from SOFCO partners and two were from other office-based companies to gain better representation of the office sector. An email with a brief description of the study was sent to all 11 invited informants, of which one SOFCO partner and one external did not respond, despite reminders. The remaining nine informants agreed to participate and received detailed written and oral information regarding the study, and provided written informed consent before data collection (40). Two informants were from the same large company, of whom one was responsible for workplace health promotion and the other one for the benefit and rewards platform that included wellness allowance. Therefore, the final sample was comprised of nine informants from eight companies, five women and four men, with positions of country manager, human resources manager, health and wellbeing specialist, or benefit and compensation manager.

### 2.3. Data collection

Data were collected through individual semi-structured interviews *via* digital video meetings on Zoom or Microsoft Teams platforms, between May and December 2021 during the COVID-19 pandemic. Informants attended from their home offices or workplaces during working hours. The interviews focused on the type and access to workplace health promotion that each company provided for its employees and covered the following topics: access and usage of wellness allowance, availability of other workplace health promotion services, management of workplace health promotion, visions and possible barriers to workplace health promotion, and promotion of active transportation. Interviews lasted on average of 40 min, were audio-recorded externally, and transcribed verbatim. In addition, quantitative data from each company were collected regarding the total number of employees and the number of office workers. Also collected from the previous calendar year were: the proportion of employees taking advantage of the wellness allowance, the maximum amount of money that an employee could spend on wellness allowance, the potential total cost for wellness allowance, the actual cost of wellness allowance, and the cost for other workplace health promotion services. The data were from the whole company, including office-, manual-, and sales-oriented workers since data were not available for office workers only.

### 2.4. Data analysis

**Figure 1:**
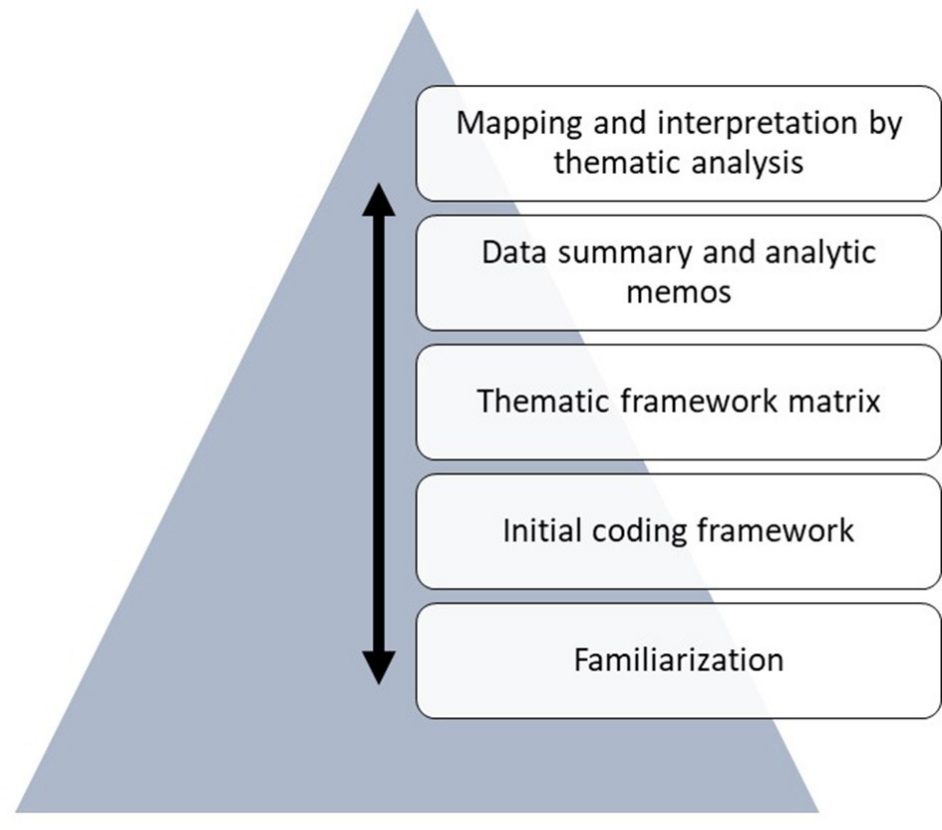
Illustration of the hierarchy and the flexible workflow between the stages of the framework analysis procedure, moving back and forth.

The framework method was used to analyze the qualitative data in a flexible and systematic way (41), based on structured coding and an analytical framework. The analysis followed the procedure described by Gale et al., with a primarily deductive approach (42), see Figure 1. After reading the transcripts independently several times (O.HU), an initial coding framework was developed based on preliminary data and presented for the other author (S.T, A.T, K.B). Excel spreadsheets were created, incorporating codes and column headings and company informant ID numbers (1–9) in rows, resulting in a thematic framework matrix. The matrix was filled with quotes and data relevant to each informant and the emerging codes by the first author. During the comparison with new data, the framework was edited and sorted to enable the introduction of new codes and allowed for the removal of other codes that became unessential. Thematic codes were developed as analytic memos describing the codes for the development of sub-themes and themes (O.HU), and the result was produced in collaboration with all authors (OHU, S.T, A.T, K.B) based on thematic analysis by mapping and interpreting the final matrix (see Table 1). Previous iterations of the coding framework matrixes were stored for transparency. Data saturation occurred when no new themes emerged from the data, and no further data collection was warranted. Quantitative data were organized in excel spreadsheets coded by company informant ID letters (A to H) and presented in Table 2 without ID numbers to ensure confidentiality.

**Table 1:** Extracts of the development of themes and sub-themes.

**Code**	**Quote**	**Theme code**	**Sub-theme**	**Theme**
Purpose health	“We simply focus on health, if you have a healthy body and mind, you will feel better at work. We offer a holistic perspective, and we try to implement it in the best way possible.”	To promote health and productivity	To promote health, productivity, and attractiveness	Reasons and ways to implement workplace health promotion
Purpose attractiveness	“To become a more attractive employer and to improve the employee value proposition, with desire to increase the motivation for workplace health promotion.”	To be an attractive employer		

**Table 2:** Descriptive data of the total number of employees and the number of office workers at each company, respectively, as well as usage and costs for wellness allowance and other workplace health promotion for the whole company, regardless of type of work description, for the year of 2020 expressed in Euro.

**Company ID letter**	**A**	**B**	**C**	**D**	**E**	**F**	**G**	**H**
Total employees (*n*)	8,540	4,816	4,500	389	72	50	14	6
Office workers (*n*)	4,560	400	3,800	389	72	50	14	6
Usage of wellness allowance (%)	55	29	55	84	59	-	80	NA^a^
Maximum amount of money that an employee may spend on wellness allowance	310	270	270	450	450	270	310	NA^a^
Total potential cost if all employees used 100% of their wellness allowance	2,600,000	1,200,000	1,200,000	175,000	31,000	13,000	4,300	NA^a^
Actual cost for wellness allowance	1,430,000	175,000	650,000	120,000	8,700	-	3,500	NA^a^
Other workplace health promotion efforts and recourses	-	850,000	4,700,000	17,500	87,000	-	-	1,600
Benefit and reward platform	Yes	No	Yes	No	No	No	No	No

a NA, Data not applicable.

## 3. Results

Table 2 describes the number of employees, usage, and costs for wellness allowance and other health promotion activities during the year 2020. The number of employees per company range from 6 to 8,540. The companies had a total of 18,387 employees, of which 9,291 were office workers. Only one company did not provide a wellness allowance to its employees. Among the rest, the lowest wellness allowance was 270 Euro per employee/year, and two companies allowed the maximal amount of 450 Euro. The potential cost, if all employees used their maximal amount of wellness allowance, would range between 4,300 Euros for the smallest company and 2.6 million for the largest company. However, the actual cost of wellness allowances ranged between 3,500 and 1.4 million Euros. The proportion of employees who took advantage of the wellness allowance ranged from 29 to 84%. Five companies provided data on the total cost for other health promotion activities per year in addition to wellness allowance, ranging from 1,600 Euro for the smallest company to 4.7 million Euros for the largest company. Two of the eight companies used an external benefit and reward platform to provide wellness allowance and other services. Shown below are results from the interviews with company informants responsible for the workplace health promotion. The analysis resulted in 23 different codes and thematic codes, 10 sub-themes, and three themes.

### 3.1. Reasons for implementation and management of workplace health promotion

#### 3.1.1. To promote health, productivity, and attractiveness

Employees are described as an essential resource, and the companies have a zero vision regarding work-related ill health. The employer encourages the employee to be and remain healthy to cope with work, be productive, and experience a high degree of work-life balance.

“*We want to promote healthy behaviors; we know it is good to be physically active. It is good for the individual, and by that, the employee becomes more productive and a healthier coworker in the long run*.” *(ID1)*

The companies' benefits and rewards are described as a way to be attractive in the labor market. By providing the right and sought-after benefits, the companies want to attract and retain top talent employees and the wellness allowance is described as a minor but important factor.

“*We want to be an attractive employer, branding, but mainly because we know that it affects people's ability to perform and be healthy.” (ID8)*

#### 3.1.2. Management of wellness allowance

Wellness allowance is managed through receipt accounting, either by employees sending their pre-paid receipt to the department for human resources or in a digital real-time operating system. Two companies provide the wellness allowance by an external company in the form of a benefit and rewards platform. Companies that offer wellness allowances follow the regulations set by the Swedish Tax Agency (15), and policies are up to date and based on market prices of gym memberships and other activities.

“*Through the benefit and rewards platform and externally by receipt accounting.” (ID5)*

“*We always refer to the tax agency regarding what you can use it for and what you cannot use it for.” (ID2)*

#### 3.1.3. The individual responsibility

Employees often have a flexible work schedule, trust-based working time, and a wellness hour for physical activity during working hours. Still, employees mainly take advantage of the wellness allowance outside regular working hours. It comes down to the individual employee or in dialog with the closest team manager if the employee can use her wellness allowance activities or perform other workplace health promotion services during working hours.

“*Primarily outside office hours, on some occasions, people have exercised during lunchtime or similar situations, but primarily outside office hours, when it comes to the wellness allowance.” (ID4)*

### 3.2. Facilitators for physical activity

#### 3.2.1. Activities purchased by employees or financed by the companies

The wellness allowance is primarily used to buy a membership at a gym, personal training, training programs, paddle tennis, or access to a swimming facility. It is also used for summer or winter activities, such as golf or ski-passes. Companies also report that an increased amount of money for wellness allowance to around 270 Euros is associated with increased usage.

“*Primarily gym memberships. I do not have exact statistics. I have problems finding it. But what I can see from the receipts that I have access to it is memberships at a gym, swimming, and yoga, which are the three primary activities. We do also get receipts for lift passes from ski resorts and other activities that it can be used for.” (ID4)*

Collaborations with external health promotion companies are common for providing health services, activities, or fitness-facilities for employees. The most common collaboration is with a local gym, personal trainer, companies that provide step and activity competitions, and manual therapists. These benefits can either be combined with the wellness allowance, purchased separately, or are provided for free to the employees. Activities are provided regularly, once or twice a year, or as one-time-only events.

“*Yes, all our suppliers are external. We have one company that provides massages at the workplace, then we have an exclusive deal with a gym to get the best price for our employees.” (ID1)*

Independent sport clubs organized within the company are also common. The companies often support the sport clubs by financial contributions or customized corporate apparel for events. However, the primary financing is through membership fees, and the employees run the association. This allows the employees to participate in different sporting activities during working hours or in their free time.

“*We have a very strong sport association where almost half of our employees are members. There are several different orientations and, in each city where we have an office, there is a specific board that represents their interests.” (ID8)*

#### 3.2.2. The employee initiative drives workplace health promotion

Newsletters and the company intranet are used to inform and communicate about workplace health promotion strategies and events. Employee initiative and engagement are essential factors regarding the amount of money the companies provide in wellness allowance and which workplace health promotion services the companies provide.

“*Yes, it is through our website, our intranet, then occasionally we try to advertise in our newsletter or in social media, so we try to reach out with information.” (ID1)*

“*We got input from the employees about what they can't do and what they want to do.” (ID4)*

#### 3.2.3. Physical activity and office ergonomics at work

Occupational health care is used for health-related problems and advice. All employees are provided height-adjustable desks for working while standing, ergonomic tools such as chairs, and keyboards. Employees are encouraged to be physically active during the workday, and two companies provide exercise equipment at the workplace, such as physioballs, rubberbands, pull-up bars, yoga mats, and bikes.

“*Sedentary behavior is not a problem, with height-adjustable tables, exercise bikes, balls and yoga mats, and other stuff. Just because the small daily activities are the most important in our opinion.” (ID7)*

“*A lot of planning went in to constructing this activity-based office. There was a lot of work to find out employee wishes regarding the workplaces and so on, as well as ergonomic workplaces, equipped with the best technology and all of those things.” (ID5)*

Four companies organize activities such as jogging, tai-chi, yoga, or group fitness workouts 1–4 days a week. Almost all companies implement walking meetings to get outdoors and promote movement during the day. Also, there are prompts, nudging and activity competitions to promote everyday physical activity. The companies that provide workplace health promotion services in-house have their facilities available during and after working hours with personal trainers for exercise advice and support. All companies except one have changing rooms and showers for both genders, and the ability to store personal belongings and sportswear adjacent to the office.

“*We have implemented various small activities around elevators and stairs to promote physical activity, take the stairs instead of the elevator, and similar things.” (ID4)*

“*We provide running classes during lunch breaks once a week, and we also have a personal trainer once a week; he provides group-based exercise classes that employees can join. We also have a yoga class once a week.” (ID2)*

“*We book meetings where we talk digitally or in real-time and walk outdoors, preferably in nature when nature is close by so that you don't have to sit still all day.” (ID7)*

#### 3.2.4. Active transportation

Active commuting to and from work is promoted by providing accessibility, security, and convenience for the employees. A few companies provide secure bicycle storage indoors, and two companies provide the possibility of borrowing bikes.

“*Good bicycle functionality means that you can store your bike in a secure manner. You can lock it in a bike stand or have it in a closed room with surveillance cameras, and we provide both. Then you always have the possibility to shower, which also simplifies life after exercise. It goes hand in hand with the gym. Inside the latest office we constructed, we built solitary showers and storage in connection with the working space, which makes it easy to shower when you have exercised or commuted by bike to work, or to change clothes on the way home.” (ID6)*

### 3.3. Visions and possible barriers

#### 3.3.1. Healthy habits as part of everyday office work

There is a vision to reach a broader range of employees, not only employees who already use their wellness allowance. Physical activity and healthy habits need to become part of everyday office work. Thus, managers and teams should be aware of the importance of physical activity promotion during working hours.

“*I believe that it is part of the company leadership to work with health and wellness. Companies make it easy for themselves when they say, we offer wellness allowance and provide height-adjustable tables, then they are done… But that is not enough. It's about living it, thinking about it, and inspiring the employees.” (ID7)*

Informant (ID8) believe that they can reach a broader range of employees by solving the employee's work-life balance constraints related to workload, pension, or family life. Other strategies and visions proposed were implementing more workshops, education, seasonal campaigns of various physical activities, easier access to healthy food, and exercise at the office.

“*An employee may need bags with groceries or homework help for their kids or continue with lunch- or public transportation subsidy. How can we adapt to the employee's needs? As we all know, the wellness allowance only reaches those who already are physically active. We want the employees to have greater freedom of choice; depending on where you are in life, you should be able to adapt and get the most out of our benefits. It is a good first step to try a new benefit. It might become an eye-opener for something different, and we try to avoid promoting high-intensity exercise. Even though we may not reach the couch potatoes who may never get out of the sofa, we may reach those who periodically exercise in a better way.” (ID8)*

#### 3.3.2. Barriers regarding wellness allowance

The one company (ID7) that currently does not provide wellness allowance reports that the main barrier is inequity between offices in different countries, where they cannot provide the allowance.

“*We are an international company, and wellness allowance is not provided across the world. On the other hand, it hasn't been on the agenda; it is next to nothing in terms of the company budget. We have talked about it, but to introduce it in all the countries we operate in is a process.” (ID7)*

There were no barriers regarding administration and receipt accounting. Only minor things are described on an employee level, not being digital enough to handle the benefit and rewards platform or not being aware of the regulations set by the Swedish Tax Agency, which can lead to purchases for which the employee is not compensated.

“*No, not that I am aware of. We use an intuitive real-time operating system for financial expenses and management of wellness allowance. So, from my point of view, I cannot imagine that there is anyone who experiences any barriers.” (ID3)*

#### 3.3.3. Barriers regarding workplace health promotion

Company culture was the main barrier, primarily related to distrust between managers and employees in relation to financial interests and time. Focusing on economic growth in internationally listed companies overshadows the focus on employee health and wellness. Other barriers were workforce culture and traditions that result in less healthy activities, such as sedentary meetings with confectionery and sweets or after work at a bar.

“*One aspect is time; as a listed company, financial interests go before employee wellness. Then there is the question of company culture. You may not be allowed to leave to take a walk or exercise during working hours. If the culture does not allow that or if there is distrust among coworkers, then it could be a barrier.” (ID8)*

Informant (ID4) also highlights that some office workers cannot leave whenever they want, for example, telephone operators who are restricted to their desks, working in a more traditional office landscape. Further, on the more structural side, the lack of accessible in-house exercise facility could be seen as a possible barrier to easy exercise during working hours.

“*Telephone operators are highly restricted and controlled during their working day. They do not have the possibility to leave for a wellness hour; they are very time controlled.” (ID4)*

“*Where we previously had the office, everything was in-house, but when we moved, external companies started to deliver fitness facilities, which means very little is available in the building. This means that it takes a few extra minutes to get to green areas, getting to the gym, which can be a barrier for employees. I know that many people say that it has become a threshold that did not exist before.” (ID8)*

## 4. Discussion

Company informants reported that all office employees at the eight private companies in the present study have access to health services, fitness facilities, activity competitions, or wellness allowance or a combination of them. The usage of wellness allowance ranged from 29 to 84%, and the amount of money allowed ranged between 270 and 450 Euro per employee/year. The companies reported an actual total cost for wellness allowance ranging between 3,500 and 1.4 million Euros during the year 2020. The purpose of providing workplace health promotion was to maintain health and productivity among employees and employee branding. The most common reported facilitators for physical activity were easily accessed fitness facilities, convenient bike storage, and changing rooms. Common barriers were outsourced fitness facilities, traditions, and distrust among managers and employees. The study also found that employee initiative and engagement is important for the development of workplace health promotion.

In line with previous studies, companies in this study use several different strategies to decrease sedentary time at the workplace, such as physically active workstations, ergonomic workstations, sociocultural and environmental factors, motivational materials, health education, and expert consultation (29, 30, 33, 34, 36, 37). The companies also worked with individual-level factors and life-friendly solutions that can facilitate or hinder physical activity, such as flexible work schedules, targeted information dissemination, personal training, and creating opportunities for employee engagement. These are essential strategies, as research has shown that employees' perceptions of organizational support and involvement affect participation in workplace health promotion (43).

Four of the companies have a wellness allowance usage in line with the average usage of the Swedish working population of about 60–70% (13, 14, 16), and only two campiness reported a higher usage than the national average. This difference could be due to company size and the mix of employees. For example, companies in this study with the lowest usage were larger and had more manual- and sales-oriented workers as compared to the smaller companies with only office workers. It could also be due to gender distribution, where men are less likely to use their wellness allowance (13).

Considerable economic resources are devoted to workplace health promotion. However, there are differences among the companies, such as the amount of wellness allowance provided and access to a benefit and rewards platform. This variation may create possible inequalities in workplace health promotion, and consequently, in the usage of wellness allowance. Previous studies have found that out-of-pocket pre-payments for financing wellness allowance might be ineffective regarding employee usage of wellness allowance (39). This is however not seen in our study, which could be due to contextual differences between sectors, workplaces, and employee incomes that this study did not cover.

It is well-established that a physically active lifestyle during and after working hours is beneficial for the individual and the company (7, 17, 19, 20). However, employee branding was mentioned as critical for providing workplace health promotion. Nowadays, companies do not solely reward their employees by salary; they also offer health insurance policies, financial advice, workshops, and food that attract, motivate, and retain the company's current and future potential employees (44). Companies in our study pay close attention to their employees to provide the right and sought-after benefits related to physical activity such as wellness allowance, and fitness facilities (45). The companies' visions are to reach a broader range of their current employees by providing easier access to physical activity, knowledge about healthy habits, and more life-friendly solutions such as the provisioning of grocery bags, which free up time for physical activity. Research has shown that the success of work health promotion implementations depends not only on the structure of the policies and intervention provided but also on organizational involvement and a culture of health (9, 43, 46). To successfully reach all those currently employed at the company, the whole organization needs to be involved by providing managers with the proper perspective, training, and resources concerning workplace health promotion related to physical activity (28, 38, 39). Managers also needs to increase social support to improve employee self-efficacy, attitudes (21), behavioral skills, and motivation to maintain behaviors related to physical activity (23). This is further supported by the American workplace health administration survey that highlights that lack of leadership support and workplace health promotion expertise hinders the adoption and continuation of workplace health promotion efforts (47). By truly adopting workplace health promotion on an organizational level, companies can focus more on eliminating barriers and creating facilitators for physical activity to reach all employed at the company, as demonstrated to some extent in this study.

The present study identified that the amount of money provided needs to be in line with the market prices of memberships and activities. Usage of a benefit and reward platform that removes out-of-pocket pre-payments, and a minimum wellness allowance of 270 Euros are important facilitators to increase usage. Further, the usage of wellness allowance in a social context such as paddle tennis, golf or group exercise organized with coworkers and friends are important for increased usage based on social support. It was also confirmed in the present study that the office setting and workplace health promotion in-house are essential for physical activity during working hours, which is consistent with previous research (21, 24, 25). The main barrier related to workplace health promotion and wellness allowance was insufficient dissemination of information.

Workplace health promotion in Sweden is justified by beneficence with emphasis on voluntary participation. Though, commercial, and economic interests also affect implementation. Supporters of workplace health promotion often describe improved employee health and reduced employer cost as a “win-win” proposition. However, the situation is much more complicated and raises concerns about employer dominance overriding employee autonomy and the potential for invasion of privacy. In the context of this study, little information is available on how office employees perceive workplace health promotion programs. Previous studies have demonstrated that employees in general are positive toward workplace health promotion, but employees might prefer to keep private life and work separate, arranging health promoting activities by themselves (48). The moral atmosphere of the organization can also have negative consequences for employees if there are strong norms that define which types of bodies are recognized as healthy, fit, or viable, and which are not (49). This affects employee recruitment by favoring the fit and healthy, resulting in discrimination and exclusion of individuals (50).

This study provides an insight into how wellness allowance and workplace health promotion can be used to facilitate physical activity among office employees in private companies. Although workplace health promotion offers are common in Sweden, this study is one of the few that explores facilitators and barriers for promoting physical activity in an office context. Knowledge of modifiable workplace health promotion polices from this study will help companies develop healthy offices in the future. If the results are to be transferred to a broader population, further research should include more diverse companies, e.g., office workers from the public sector. The pandemic has resulted in increased sedentary behaviors and changes in physical activity levels among the Swedish population (51), emphasizing the value of studies of how the pandemic might have affected workplace health promotion policies.

### 4.1. Strengths and limitations of the study

The first author collected, organized, and analyzed all data in discussion it with the co-authors to ensure trustworthiness. Collaboration with the co-authors enabled reviewing of codes, framework matrixes, category labels, and our interpretations. The framework analysis provided a base for collaboration during the ongoing COVID-19 pandemic and teleworking that allowed the research team to produce results in transparent and accessible ways. To obtain in-depth descriptions of how workplace health promotion is managed, individual interviews were used to study the context and code of conduct outside policy documents. Consequently, we asked the informants about their own procedures and actions, which enriches the understanding of implementation, barriers, and facilitators for physical activity in an office context.

A limitation to this study is the lack of investigation of individual receipt accounting and policy documents, that would have revealed more information about: what the wellness allowance is used for, actual costs for wellness allowance, and workplace health promotion specifically for office workers only. Moreover, we did not collect data on socio-demographic factors of each employee. Previous studies have shown that socio-demographic factors may impact participation in workplace health promotion, where manual- and sales-oriented workers, individuals residing in smaller cities or employees with low income may face challenges in participating or financing wellness allowance due to out-of-pocket payment (13, 39). Due to the ongoing COVID-19 pandemic, digital interviews were used, limiting the researcher's ability to observe the behaviors and body language of the informants. We also lacked the possibility of visual context of the built environment. Two companies chose not to participate in the study, and it is possible that these informants had limited interest or knowledge about workplace health promotion and chose not to participate for that reason. It may lead to favoring particularly interested informants and companies with well-developed workplace health promotion. The transferability of the study is applicable to settings with similar organizational conditions as is the case for several of the companies included in the study, i.e., new ways of working in private activity-based digital offices located in big cities, often in more temporary and collaborative projects (52). However, some of the larger companies are more heterogeneous, and despite the limited number of informants interviewed in the present study, they provided information on wellness allowance that is representative for different types of employees and occupations in various geographical regions across the country. Hence, the results are sufficient to illustrate the use of wellness allowance and health promotion in the office business in Sweden.

## 5. Conclusions

This study contributes new knowledge of facilitators and barriers to physical activity during and after working hours. Companies have several strategies to promote physical activity to maintain employee health and attract and retain top talents. The study demonstrates that dissemination of information, convenience and easy access inside the office are key facilitators for promoting physical activity. Also, physical activity and healthy habits need to become part of everyday office work. The practical implications of this study indicate that wellness allowances must be in line with market prices. Managers need to be aware of company culture and increase flexibility regarding working hours and the built environment. Moreover, they need to offer life-friendly solutions to address work-life balance constraints that hinder a healthy lifestyle. This study also highlights the importance of employees' engagement in developing and improving workplace health promotion.

## Data availability statement

The raw data supporting the conclusions of this article will be made available by the authors, without undue reservation.

## Ethics statement

The studies involving human participants were reviewed and approved by Ethical Review Board of Uppsala for Studies Involving Humans (Dnr: 2021-02309). The participants provided their written informed consent to participate in this study.

## Author contributions

KB, ST, and OHU: conceptualization. OHU: methodology, investigation, writing—original draft preparation, and visualization. OHU, KB, ST, and AT: formal analysis and writing—review and editing. KB and ST: resources, project administration, and funding acquisition. KB and OHU: data curation. KB, ST, and AT: supervision. All authors have read and agreed to the published version of the manuscript.
